# The clinical impact of donor against recipient HLA one way mismatch on the occurrence of graft versus host disease in liver transplantation

**DOI:** 10.1038/s41598-022-24778-2

**Published:** 2022-11-25

**Authors:** Sang Jin Kim, Sunghae Park, Jinsoo Rhu, Jong Man Kim, Gyu-Seong Choi, Jae-Won Joh

**Affiliations:** 1 Department of Surgery, Korea University College of Medicine, Seoul, Republic of Korea; 2 Division of Hepatobiliopancreas and Transplant Surgery, Korea University Ansan Hospital, Ansan, Republic of Korea; 3 Department of Surgery, Samsung Medical Center, Sungkyunkwan University School of Medicine, 81 Irwon-Ro, Gangnam-Gu, Seoul, 06351 Republic of Korea

**Keywords:** Liver, Outcomes research

## Abstract

Graft versus host disease (GVHD) after liver transplantation (LT) is a rare, fatal disease. This study aimed to evaluate the risk factors of GVHD after LT including the human leukocyte antigen (HLA) donor-recipient relationship after LT. LT recipients, who underwent HLA typing together with donors, were included in the study. The donor against recipient (D → R) one-way mismatch of HLA loci was evaluated. HLA relationships, along with basic characteristics, were analyzed as variable factors of GVHD, graft survival, and patient survival. A total of 994 living donor LT (LDLT) and 393 deceased donor LT (DDLT) patients were included. Nine patients had suffered GVHD, four LDLT with D → R one-way at three loci, one LDLT without D → R one-way at three loci, and four DDLT without D → R one-way at three loci. Four (57.1%) of seven LDLT patients, with D → R one-way mismatch at three loci, developed GVHD. D → R one-way mismatch at three loci was related to high GVHD incidence (HR 787, *p* < 0.001, multivariate). D → R one-way mismatch at three loci was related to graft failure and patient death (HR 9.90, *p* = 0.020 and HR 12.8, *p* < 0.001, respectively, multivariate). Only one GVHD without D → R one-way mismatch at three loci, survived despite receiving multiple modalities including tumor necrosis factor-alpha inhibitors. D → R one-way mismatch at three loci was significantly related to GVHD incidence after LT.

## Introduction

Graft versus host disease (GVHD) after liver transplantation (LT) is a condition that occurs when T lymphocytes derived from the donor recognize recipient’s cell as foreign body leading to an immune reaction against recipient organs^1^. Although the condition is rare, the consequences are fatal, reaching more than 75% mortality^2^. Owing to the rarity and severity of GVHD in LT, the risk factors, treatments, and patient courses of GVHD have not yet been established.

There seems to be a number of risk factors that contribute to the development of GVHD in LT, but how these contribute remains unclear. These include diabetes mellitus (DM)^3^, differences in recipient and donor ages of more than 20 years^4,5^, closely related human leukocyte antigen (HLA) compatibility^6,7^, and immunotherapies^5^. However, more information is needed regarding risk factors for GVHD.

GVHD in LT usually develops approximately 28 days after transplantation, with a skin rash (92%), cytopenia (78%), and diarrhea (65%) as the most common symptoms^2^. Approximately 73% of GVHD patients die within 6 months due to infection, multi-organ failure and gastrointestinal bleeding. High-dose steroids, cessation or decrease of immunosuppressants, or anti-thymocyte globulin (ATG) therapy are commonly used methods for treatment without established evidence.

This study was designed to analyze the risk factors for GVHD after LT, including human leukocyte antigen (HLA) compatibilities, at a single high-volume center.

## Patients and methods

### Patient inclusion

Both living and deceased donor LTs performed at the Samsung Medical Center (Seoul, Korea) were reviewed for inclusion in the study. Recipients and donors without HLA typing were excluded from the study. Considering the mean onset time of GVHD, patients with a follow-up period of less than 6 months were also excluded, except those who died or received re-transplantation within 6 months due to other specific events. Since HLA typing for LT started in 2009, LT cases before 2009 were excluded. Relationships, especially mismatches between donor and recipient’s HLA types, were reviewed along with the relationship between donors and recipients. Other preoperative parameters, such as recipient age, sex, body mass index, Child-Turcotte-Pugh score and Model for End-stage Liver disease (MELD) score, donor age, and sex, were evaluated as risk factors. Underlying diseases in recipients such as hypertension (HTN), DM, hepatitis B virus (HBV), hepatitis C virus (HCV), hepatocellular carcinoma (HCC) and ABO blood type incompatibilities between the donor and recipient were evaluated. The informed consent was obtained from all subjects and/or next of kin including deceased patients. The informed consent was also obtained from all subjects and/or their legal guardian(s)/next of kins for publication of identifying information (Case #1 to #10 in supplementary information 4). This study was conducted in accordance with the Declaration of Helsinki, relevant guidelines and regulations, including the de-identification of protected health information and the exclusion of prisoners' organ procurement. This study was retrospectively designed and was approved by the Institutional IRB of Samsung Medical Center (IRB Number 2021-06-014-001).

### Basic immunosuppressive agents

As induction agents, interleukin-2 receptor monoclonal antibody (basiliximab, Novartis Pharmaceuticals, Basel, Switzerland) and high-dose steroid (methylprednisolone starting with 500 mg intravenous for two days) were injected. To maintain of immunosuppression, calcineurin inhibitors (usually tacrolimus), anti-proliferative agents (mycophenolate mofetil or mycophenolate sodium), and steroids were administered. When the patient and donor had incompatibility with the ABO blood type, anti CD20 agent (rituximab) was administered at 2 weeks before transplantation followed by preoperative plasmapheresis. For patients who were at high risk of HCC recurrence, mammalian target of rapamycin inhibitor (sirolimus, Pfizer) was added at 1 month after transplantation with lowering serum drug level of tacrolimus.

### Evaluation and diagnosis of GVHD

GVHD usually develops approximately 1 month after LT. When a patient develops symptoms such as skin rash, cytopenia or diarrhea, the transplantation team should be cautious about the possibility of GVHD. If the symptoms were sustained without other explainable reasons or two or more symptoms were present, pathological biopsy, such as skin or colonoscopic biopsy, was performed. Bone marrow biopsy was considered only when cytopenia was the only GVHD symptom. While the basic diagnosis and decision of treatment for GVHD was based on pathological examination, some patients were tested using quantitative polymerase chain reaction (qPCR) by the targeting insertion and/or deletion (INDEL) method. We regarded donor lymphocyte macro-chimerism (> 1%) in recipient peripheral blood as confirmation of GVHD. However, we did not wait for confirmation before treating a patient with GVHD.

### HLA typing

Serologic HLA typing of HLA -A, -B, and –DR were performed using polymerase chain reaction (PCR) in both donors and recipients (each six HLA loci). “Donor against recipient (D → R) mismatch” was defined as loci within recipient’s HLA in certain loci not being present in the donor’s HLA in the same loci. “Recipient against donor (R → D) mismatch” was defined as certain loci within the donor’s HLA not being present in the recipient’s HLA in the same loci. “Donor against recipient one-way mismatch” was defined as the recipient’s HLA not being present in the donor’s HLA, while all the donor’s HLA were included in recipient’s HLA. Based on a previous study^6^, the numbers of D → R one-way mismatches were categorized as “3” and “1 or 2”. HLA homozygosity and one-way mismatch were analyzed according to donor and patient genetic relationships.

### Graft survival and patient survival

We defined “graft failure” as death or re-transplantation due to failure of the transplanted liver. The relationship between the number of HLA mismatches and graft and, patient survivals were analyzed. Risk factors related to graft failure and patient death were also analyzed.

### Statistical analysis

Student’s t-test, Pearson’s chi-square test, and Fisher’s exact test were performed to compare the characteristics of living donor LT (LDLT) and deceased donor LT (DDLT). Cox regression analysis was performed to analyze the risk factors for GVHD, graft failure, and patient death and survivals. Multivariate analysis was performed, including variables that showed a *p* value of < 0.1 in univariate analysis, along with crucial factors such as donor type. Statistical significance was defined as a *p* value of < 0.05. These analyses were performed using the IBM SPSS-24 statistical program (IBM Institute, New York, USA).

## Results

### Baseline characteristics

**Figure 1:**
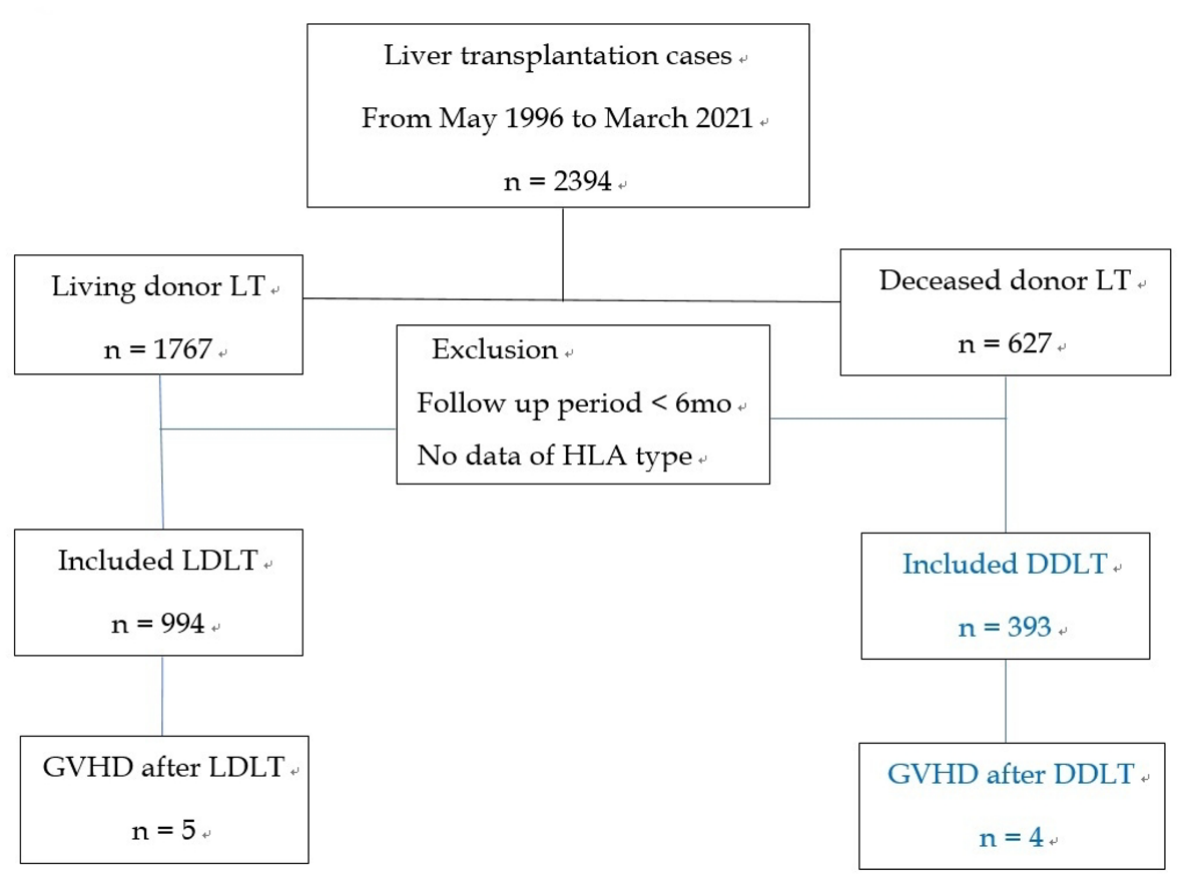
Flowchart of patient selection.

From May 1996 to March 2021, 2,394 patients received LT at the Samsung Medical Center. A total of 1,767 patients received LDLT and 627 received DDLT. After the exclusion of patients with follow-up periods of < 6 months and cases with missing HLA type (before 2009), 994 LDLT patients and 393 DDLT patients were included in the study (Fig. 1). Patient characteristics are described in Table 1. The mean follow-up period was 1193 ± 1,114 days. Mean ages of the recipients and donors were 50.0 ± 15.8 and 37.5 ± 15.0 years, respectively. Nine patients developed GVHD, including five patients with LDLT (0.5%).

**Table 1:** Patient characteristics according to donor types.

	ALL patients (n = 1387)	LDLT (n = 994)	DDLT (n = 393)	
Male, n	970 (70.0%)	735 (73.9)	235 (59.8%)	< 0.001
Age, mean	50.0 ± 15.8	51.2 ± 14.2	46.8 ± 19.0	< 0.001
Donor age, mean	37.5 ± 15.0	34.2 ± 12.7	45.9 ± 17.1	< 0.001
Age difference, mean (Recipient—Donor)	14.9 ± 18.7	19.0 ± 16.7	4.3 ± 19.3	< 0.001
Age difference > 20 years, n	721 (52.0%)	641 (64.5%)	80 (20.4%)	< 0.001
BMI, mean	24.2 ± 4.3	24.3 ± 4.0	23.8 ± 5.1	0.454
CTP, mean	8.5 ± 2.6	7.9 ± 2.5	10.3 ± 2.0	< 0.001
MELD, mean	20.1 ± 12.3	16.1 ± 10.1	30.1 ± 11.8	< 0.001
HTN, n	189 (13.6%)	140 (14.1%)	49 (12.5%)	0.429
DM, n	279 (20.1%)	198 (19.9%)	81 (20.6%)	0.772
HBV, n	780 (56.2%)	621 (62.5%)	156 (40.5%)	< 0.001
HCV, n	79 (5.7%)	56 (5.6%)	23 (5.9%)	0.898
Alcoholics, n	244 (17.6%)	149 (15.0%)	95 (24.2%)	< 0.001
HCC, n	705 (50.8%)	594 (59.8%)	111 (28.2%)	< 0.001
ABOi, n	188 (13.6%)	188 (18.9%)	0	< 0.001
Re-LT cases, n	74 (5.3%)	8 (0.8%)	66 (16.8%)	< 0.001
R → D one-way MM3*, n	10 (0.7%)	10 (1.0%)	0	0.071
D → R one-way MM3^†^, n	7 (0.5%)	7 (0.7%)	0	0.201
Follow-up days, mean	1193 ± 1114	1290 ± 1154	949 ± 965	0.020
GVHD, n	9 (0.6%)	5 (0.5%)	4 (1.0%)	0.282
Graft fail, n	137 (9.9%)	57 (5.7%)	80 (20.4%)	< 0.001
Re-LT, n	38 (2.7%)	25 (2.5%)	13 (3.3%)	0.528
Death, n	304 (21.9%)	181 (18.2%)	123 (31.3%)	< 0.001

*Recipient against donor one-way mismatch in 3 loci.

^†^ Donor against recipient one-way mismatch in 3 loci.

### HLA type relationship and GVHD risk

Among the 994 LDLT patients, 12 had a D → R one-way mismatch (Table 2). There was no D → R one-way mismatch case in DDLT. Seven patients had D → R one-way mismatch at three loci (D → R one-way MM3). All donors of these relationships had homozygous HLA types in –A,B and DR and they donated to their blood-related family (five to parents, one to daughter and one to sister). Among these seven patients, four developed GVHD (57.1%). One patient with D → R one-way MM3 had primary graft non-function and was re-transplanted with a deceased donor graft without HLA mismatch. He had a liver graft with D → R one-way MM3 for only 4 days; this patient did not develop GVHD after re-transplantation. On the other hand, none of the patients with D → R one-way mismatch at one or two loci, R → D one-way mismatch, or fully matched HLA types developed GVHD. Among other cases besides one-way mismatch or full match, the number of GVHD cases was one (0.1%) and four (1%) among LDLT and DDLT groups, respectively. All LDLT donors in the D → R one-way MM3 pairs had a homozygous HLA type (Table 3). Among the five patients who developed GVHD after LDLT, three received liver grafts from offspring with a homozygous HLA type, while one patient received a liver graft from a homozygous HLA-type mother, leading to D → R one-way MM3. The total mismatches in each LDLT and DDLT patient groups are described in Supplement 1, and each donor and recipient’s HLA type of D → R one-way MM3 cases are described in Supplement 2.

**Table 2:** HLA relationship between donor and recipient and GVHD.

	Relation of HLA matching between donor and recipient	Number of cases	GVHD incidence
Living donor liver transplantation	Donor against recipient one-way mismatch at 3 loci	7*	4 (57.1%)
	Donor against recipient one-way mismatch at 1 or 2 loci	5	0
	Recipient against donor one-way mismatch at 3 loci	10	0
	Recipient against donor one-way mismatch at 1 or 2 loci	6	0
	Full match between donor and recipient	32	0
	Cases without one-way mismatch or full match	934	1 (0.1%)
Deceased donor liver transplantation			
	Cases without one-way mismatch or full match	393	4 (1%)

*One patient with D → R one-way MM3 had primary graft non-function, received re-LT on postoperative day#4, leading to change of HLA relationship.

**Table 3:** Number of homozygous HLA loci according to relationship between donor and recipient in LDLT.

Relationship (donor)	Homozygous HLA loci	One-way mismatch in 3 loci	GVHD incidence
Offspring (n = 587)	Recipient side = 11	Recipient against donor = 10	
	Donor side = 5	Donor against recipient = 5	3
	Not homozygous = 571		1
Parent (n = 63)	Donor side = 1	Donor against recipient = 1	1
Sibling (n = 103)	Donor side = 2	Donor against recipient = 1	
	Both side = 2	Full match = 2	
Relatives in blood* (n = 25)	Recipient side = 1		
Spouse^†^ (n = 155)	None		
Other relationships^†^ (n = 61)	None		

*Relatives who are not parent, offspring, or sibling.

^†^Genetically unrelated relationships.

**Figure 2:**
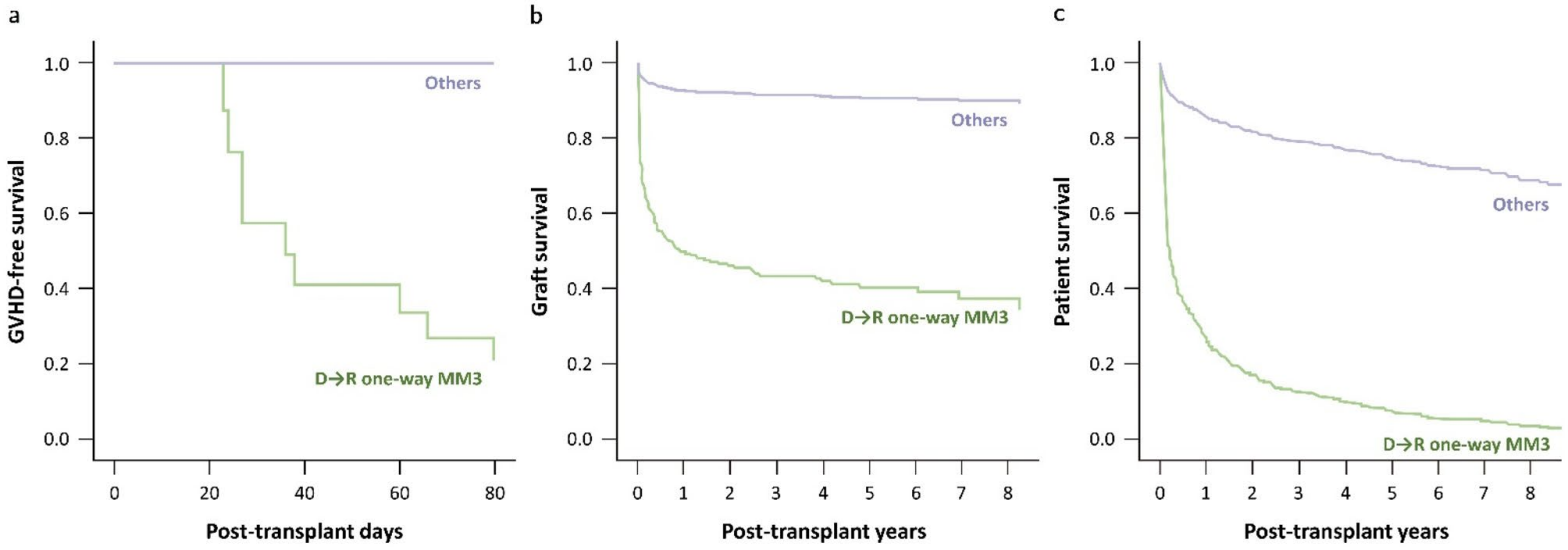
GVHD-free, graft and patient survival of patients with “donor against recipient one-way mismatch at three loci”.

Patients with D → R one-way MM3 had significantly lower GVHD-free survival, with only 53.6% survival at 60 days. A cox-adjusted survival graph is shown in Fig. 2. Risk factors for GVHD were analyzed by using the Cox regression method (Table 4). D → R one-way MM3 was significantly related to GVHD incidence in multivariate analysis (HR 787, *p* < 0.001). Re-transplantation (re-LT) was significantly related to GVHD only in univariate analysis but not in multivariate analysis (HR 5.85, *p* = 0.028, univariate). Other factors, such as donor type (*p* = 0.232), recipient age (*p* = 0.467), age difference between donor and recipient (*p* = 0.409), MELD score (*p* = 0.171), DM (*p* = 0.324), HBV (*p* = 0.162), HCV (*p* = 0.618), and alcoholic liver disease (*p* = 0.213) were not significantly associated with GVHD in univariate analysis. The separate risk factors of GVHD in each LDLT and DDLT is described in Supplement 3. However, the statistical analysis could not draw the appropriate result of influence of D → R one-way MM3 on GVHD in LDLT, due to very small number of GVHD and D → R one-way MM3 cases. There was no specific risk factor which showed statistical significance except that only re-LT showed tendency of high GVHD risk in DDLT.

**Table 4:** Risk factors for GVHD, graft failure, and patient death.

Risk factors	GVHD incidence				Graft failure				Patient death			
	Univariate analysis		Multivariate analysis		Univariate analysis		Multivariate analysis		Univariate analysis		Multivariate analysis	
	HR (95% CI)	*p* value	HR (95% CI)	*p* value	HR (95% CI)	*p* value	HR (95% CI)	*p* value	HR (95% CI)	*p* value	HR (95% CI)	*p* value
DDLT	2.23 (0.60–8.30)	0.232	6.89 (0.65–73.1)	0.109	4.08 (2.90–5.73)	< 0.001	2.77 (1.79–4.27)	< 0.001	2.00 (1.59–2.51)	< 0.001	1.64 (1.22–2.22)	0.001
D → R one-way MM3	207 (54.4–790)	< 0.001	787 (86.6–7148)	< 0.001	5.06 (1.25–20.5)	0.023	9.90 (2.37–41.4)	0.002	9.26 (3.80–22.6)	< 0.001	12.8 (5.20–31.5)	< 0.001
Donor age	1.00 (0.96–1.05)	0.888			1.02 (1.01–1.03)	< 0.001	1.00 (0.98–1.02)	0.738	1.01 (1.01–1.02)	< 0.001	1.01 (0.99–1.02)	0.433
Age difference (Recipient—donor age)	1.00 (0.94–1.04)	0.903			0.98 (0.97–0.99)	< 0.001	1.00 (0.98–1.02)	0.790	0.99 (0.99–1.00)	0.034	1.00 (0.99–1.02)	0.532
Age difference ≥ 20 year	1.79 (0.45–7.17)	0.409			0.40 (0.28–0.57)	< 0.001	0.77 (0.52–1.13)	0.182	0.69 (0.55–0.86)	0.001	0.87 (0.65–1.17)	0.350
Age	1.02 (0.97–1.08)	0.467			0.99 (0.98–1.00)	0.036	1.00 (0.98–1.01)	0.877	1.01 (1.00–1.02)	0.036	1.01 (1.00–1.02)	0.214
Male	0.84 (0.21–3.36)	0.805			0.74 (0.52–1.04)	0.085	1.06 (0.73–1.53)	0.772	1.11 (0.86–1.43)	0.417		
BMI	0.93 (0.80–1.08)	0.329			0.98 (0.94–1.02)	0.279			0.99 (0.97–1.02)	0.678		
CTP score	1.12 (0.87–1.44)	0.395			1.14 (1.07–1.21)	< 0.001	0.94 (0.84–1.05)	0.291	1.04 (1.00–1.09)	0.061	0.94 (0.87–1.01)	0.080
MELD score	1.04 (0.99–1.09)	0.171			1.04 (1.02–1.05)	< 0.001	1.02 (0.99–1.04)	0.193	1.02 (1.01–1.03)	< 0.001	1.02 (1.00–1.04)	0.032
HTN	1.84 (0.38–8.85)	0.447			1.07 (0.66–1.74)	0.783			1.35 (1.00–1.82)	0.054	1.28 (0.93–1.76)	0.130
DM	2.01 (0.50–8.03)	0.324			0.87 (0.56–1.35)	0.524			1.26 (0.97–1.64)	0.086	1.12 (0.85–1.48)	0.436
Re-LT	5.85 (1.22–28.2)	0.028	5.21 (0.76–35.8)	0.094	3.54 (2.18–5.76)	< 0.001	1.48 (0.88–2.51)	0.143	2.64 (1.81–3.86)	< 0.001	1.90 (1.25–2.87)	0.003
ABO incompatible	0.04 (0.00–149)	0.442			0.99 (0.60–1.62)	0.963			0.87 (0.61–1.24)	0.443		
HBV	0.37 (0.09–1.49)	0.162			0.51 (0.36–0.71)	< 0.001	0.75 (0.50–1.11)	0.152	0.83 (0.66–1.04)	0.103		
HCV	0.05 (0.00–8536)	0.618			0.99 (0.49–2.03)	0.982			1.29 (0.84–1.97)	0.244		
Alcoholic	2.42 (0.60–9.66)	0.213			1.33 (0.88–2.01)	0.182			1.13 (0.84–1.52)	0.435		
HCC	1.14 (0.31–4.23)	0.850			0.48 (0.34–0.68)	< 0.001	0.77 (0.49–1.22)	0.266	1.07 (0.85–1.34)	0.565		

GVHD, graft-versus-host disease; DDLT, deceased donor liver transplantation; D → R one-way MM3, donor against recipient one-way mismatch at three loci; BMI, body mass index; CTP, Child-Turcotte-Pugh; MELD, model for end-stage liver disease; HTN, hypertension; DM, diabetes mellitus; Re-LT, re-liver transplantation; HBV, hepatitis B virus; HCV, hepatitis C virus; HCC, hepatocellular carcinoma.

### Graft survival and patient survival

With a mean follow up of 1,193 ± 1114 days, the 5-year graft survival rates of 1,387 patients, LDLT patients (n = 994) and DDLT patients (n = 393) were 88.6%, 93.0%, and 77.5%, respectively. Patients with D → R one-way MM3 had significantly lower graft survival than the other patients (Fig. 2, *p* = 0.002). Using Cox-regression analysis, the risk factors for graft failure were analyzed. (Table 4.) DDLT (HR 2.77, *p* < 0.001) and D → R one-way MM3 (HR 9.90, *p* = 0.002) were significant risk factors for graft failure in multivariate analysis. Among the seven recipients with D → R one-way MM3, two patients who did not develop GVHD ended up with graft failure on postoperative days #4 (primary non-function patient) and #326.

The 5-year survival rates of total patients, LDLT patient and DDLT patients were 74.0%, 77.5%, and 65.1%, respectively (*p* < 0.001). Patients with D → R one-way MM3 had significantly lower patient survival than the other patients (Fig. 2). DDLT (HR 1.64, *p* = 0.001), D → R one-way MM3 (HR 12.8, *p* < 0.001), high MELD score (HR 1.02, *p* = 0.032) and re-LT (HR 1.90, *p* = 0.003) were significantly associated with patient mortality in multivariate analysis.

### GVHD patient’s clinical features and courses

**Figure 3:**
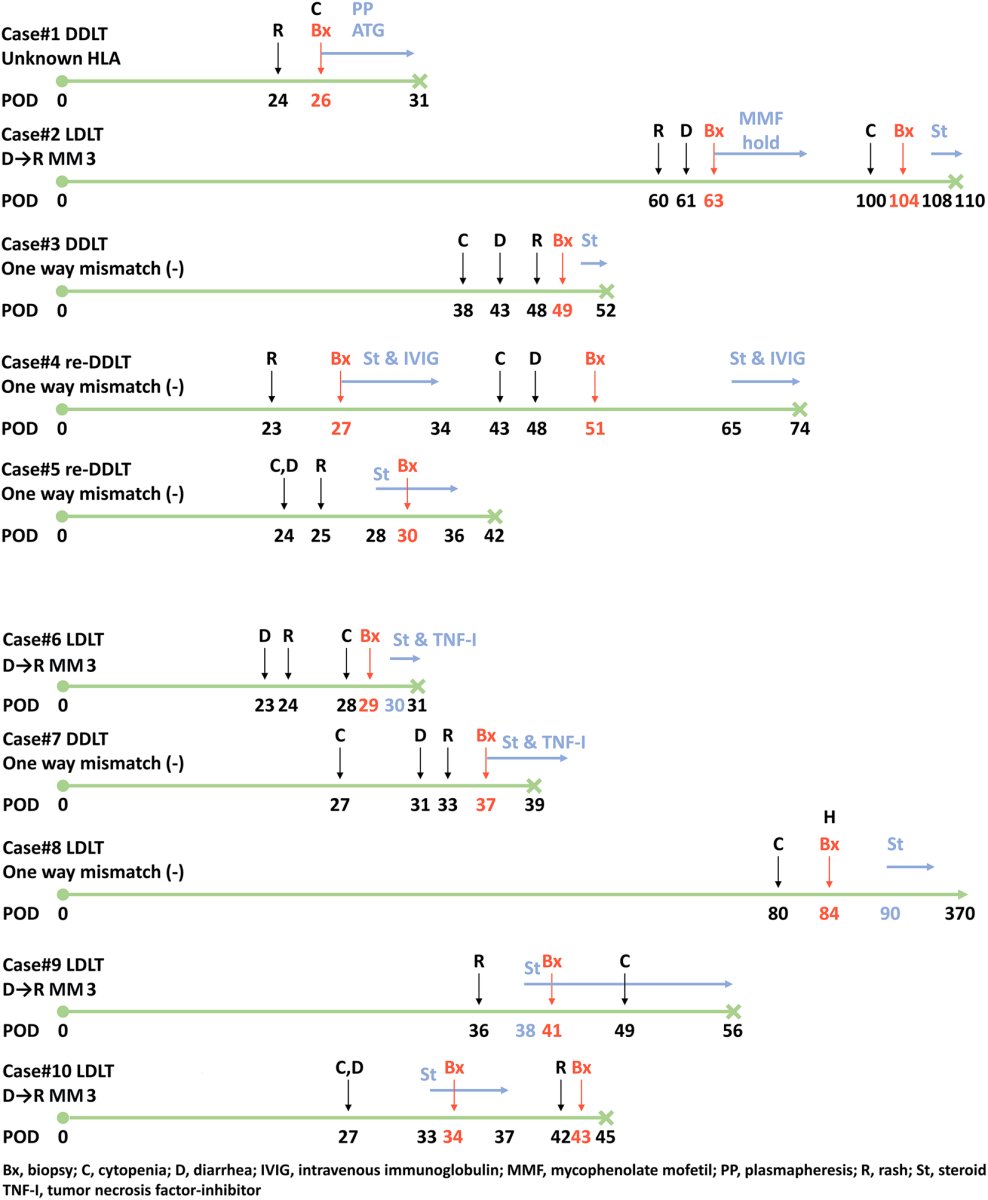
Timelines of patients with GVHD.

We added the information of one more GVHD case (case#1) that received LT in 1998 to inform the clinical features of GVHD patients as much as possible, even though the patient received LT before the transplantation center started to evaluate HLA types. Thus, characteristics, diagnosis, and treatment of a total 10 GVHD patient are described in Table 5. In contrast to the other patients, case #1 received cyclosporine, and steroid without basiliximab. The clinical course of the patients is visualized as timelines in Fig. 3. The progression and distinctive features of each case are summarized in Supplement 4 (Table 6).

**Table 5:** Characteristics and HLA types of each patient with GVHD.

Case No	Sex/age	LT type (donor relationship)	HLA type			Donor against recipient one way mismatch in 3 loci	Reason for LT	DM	GVHD first onset (post-LT days)	GVHD-related death (survival days)
			A locus	B locus	DR					
1						Unknown	LC-B HCC	(−)	24	Yes (31)
Recipient	M/49	DDLT	Unknown	Unknown	Unknown					
Donor	M/26	(unrelated)								
2						Yes	Drug induced ALF	(−)	60	Yes (110)
Recipient	F/19 F/42	LDLT	02, 33	44, 45	13, 15					
Donor		(mom)	33, –	44, –	13, –					
3						No	LC-A	(−)	38	Yes (52)
Recipient	M/53	DDLT	31, 33	35, 44	07, 09					
Donor	M/43	(unrelated)	24, –	52, 59	04, 15					
4						No	LC-C HCC Graft failure	(−)	23	Yes (74)
Recipient	M/64	Re-DDLT	30, 33	14, 58	08, 13					
Donor	M/43	(unrelated)	2, 24	27, 75	04, 09					
5						No	HCC-B Graft failure	(−)	24	Yes (42)
Recipient	M/58	Re-DDLT	02:01, 24:02	40:01, 55:02	04:03, 12:01					
Donor	M/47	(unrelated)	02, 24	07, 48	01, 11					
6						Yes	LC-B HCC	(+)	23	Yes (31)
Recipient	M/66	LDLT	24:02, 33:03	44:03, 46:01	07:01, 08:03					
Donor	F/38	(daughter)	33:03, –	44:03, –	07:01, –					
7						No	LC-A	(+)	27	Yes (39)
Recipient	M/48	DDLT	02:01, –	13:01, 35:01	11:01, 12:02					
Donor	M/24	(unrelated)	02, 11	51, 62	04, –					
8						No	HCC-B	(−)	80	No (370)
Recipient	F/68	LDLT	02:01, 11:01	35:01, 39:01	08:03, –					
Donor	M/43	(son)	02:01, 26:01	15:01, 35:01	08:03, 15:01					
9						Yes	LC-A	(−)	36	Yes (56)
Recipient	M/41	LDLT	31:01, 33:03	44:03, 46:01	08:03, 13:02					
Donor	F/19	(daughter)	33:03, –	44:03, –	13:02, –					
10						Yes	HCC-B	(−)	27	Yes (45)
Recipient	F/66	LDLT	11:01, 33:03	54:01, 58:01	04:05, 13:02					
Donor	F/41	(daughter)	33:03, –	58:01, –	13:02, –

**Table 6:** Clinical presentation and treatment of each patient with GVHD.

Case No	Sex/age	LT type (relationship)	GVHD features (onset)		Diagnostic procedure for biopsy	Steroid dose for GVHD (start day)	Other treatment for GVHD (start day)	Gap between treatment and onset	GVHD-related death (survival days)
1 Recipient Donor	M/49 M/26	DDLT (unrelated)	Rash Pancytopenia	(24) (26)	Skin (26)	mPD 40 mg daily (26)	ATG (26) Cyclo → FK (26) PP (29)	2	Yes (31)
2 Recipient Donor	F/19 F/42	LDLT (mother)	Rash Diarrhea Leukopenia Vomiting	(60) (61) (100) (100)	Colon (63) Skin (63) EGD (104)	Hydrocortisone 200 mg daily (108)	MMF hold (63)	3	Yes (110)
3 Recipient Donor	M/53 M/43	DDLT (unrelated)	Pancytopenia Diarrhea Rash	(38) (43) (48)	Skin (49)	Hydrocortisone 200 mg daily (50)	Pre-FK, MMF hold (45)	12	Yes (52)
4 Recipient Donor	M/64 M/43	Re-DDLT (unrelated)	Rash Pancytopenia Diarrhea	(23) (43) (48)	Skin (27) Colon (51)	mPd 60 mg x 7d (27) mPd 60 mg x 9d (65)	IVIG 25 mg x 2d (27) IVIG 25 mg x 3d (65) FK hold (23, re-start)	4	Yes (74)
5 Recipient Donor	M/58 M/47	Re-DDLT (unrelated)	Pancytopenia diarrhea Rash	(24) (24) (25)	Skin (30)	Hydrocortisone 200 mg x 8d (28)	FK-reduced (28)	4	Yes (42)
6 Recipient Donor	M/66 F/38	LDLT (daughter)	Diarrhea Rash Neutropenia	(23) (24) (28)	Skin (29) qPCR	mPd 500 mg (30)	TNF-α inhibitor (30) FK hold	7	Yes (31)
7 Recipient Donor	M/48 M/24	DDLT (unrelated)	Pancytopenia Diarrhea Rash	(27) (31) (33)	Skin (37) qPCR	hydrocortisone 200 mg Followed by 62 mg daily (37)	TNF-α inhibitor(38) FK hold	10	Yes (39)
8 Recipient Donor	F/68 M/43	LDLT (son)	Pancytopenia Hematochezia	(80) (84)	Colon (84) qPCR	mPd 120 mg daily (90)	FK hold (90)	10	No (370)
9 Recipient Donor	M/41 F/19	LDLT (daughter)	Rash Pancytopenia	(36) (49)	Skin (41)	Hydrocortisone 200 mg daily (38)	FK-pre hold (33)	2	Yes (56)
10 Recipient Donor	F/66 F/41	LDLT (daughter)	Diarrhea Leukopenia Rash	(27) (28) (42)	Sigmoid (34) Skin (43)	mPd 60 mg x 4d (33)	FK hold (33)	6	Yes (45)

GVHD, graft versus host disease; LT, liver transplantation; R, recipient; D, donor; DM, diabetes mellitus; M, male; F, female; LDLT, living donor liver transplantation; DDLT, deceased donor liver transplantation; TNF, tumor necrosis factor; mPd, methylprednisolone; d, days.

The recipient age ranged from 19 to 68 years with a median age of 58 years, while the donor age ranged from 19 to 47 with a median age of 41.5 years old. Five patients received livers from living donors, and the other five patients received livers from deceased donors. Among the five living donors, four donors were the son or daughter of the recipient, and one donor was the mother of the patient. Two patients received re-LT after previous LDLT. Three patients (case #6–8) were confirmed to have GVHD by both pathologic reports and qPCR assays targeting INDEL polymorphism. Only one patient (case #8) with GVHD survived for > 1 year after receiving steroid therapy. She had a relatively low degree of macro-chimerism (4.76% of the mean fraction of donor-derived deoxyribonucleic acid) compared with the other two patients (case #6, 39.68%; case #7, 78.38%). All other patients died due to GVHD even after receiving treatment with steroid, anti-thymoglobulin, IV immunoglobulin, plasmapheresis or even tumor necrosis factor-alpha (TNF-α) inhibitor. The median onset of GVHD symptoms was 27 postoperative days (range, 26–80 days), while the median day for the diagnostic procedure was 35.5 days, with the median gap between onset and diagnosis was 4.5 days (range, 0–16 days). Among the patients, seven developed all three features of skin rash, cytopenia and gastrointestinal (GI) symptom, while two did not develop GI symptoms and one patient did not show skin rash. The most frequent earliest type of GVHD symptom was pancytopenia and rash (each in four patients respectively). The median gap between first onset and treatment of GVHD was 6 days. Excluding one patient who was still alive, the median survival time was 45 days after transplantation and the median gap between onset and death was 18 days.

## Discussion

In this study, we evaluated the matching of HLA types between recipients and donors with a focus on GVHD and other outcomes. While DDLT cases had no one-way mismatch, there were seven cases of D → R one-way MM3 among LDLT cases (0.7%). Among these cases, four (57.1%) developed GVHD. All these four GVHD patients received liver grafts from donors with homozygous HLA at three loci. In fact, one of the D → R one-way MM3 cases received re-LT on the 4th day due to a primary non-functioning graft and the HLA type was changed, so it cannot be said that they had a sufficient period for GVHD expression. Therefore, with the exclusion of this case, four out of six patients (66.7%) who had D → R one-way MM3 developed GVHD. Other one-way mismatch and full-match cases did not develop GVHD. Only one case (0.1%) in the LDLT group and four cases (1.0%) in the DDLT had no one-way mismatch or full mismatch. Only one patient survived GVHD. The D → R one-way MM3 was also associated with poor graft and patient survival.

GVHD is relatively common in hematopoietic stem cell transplantation and has been widely studied with risk factors to the level of discovering HLA combination types in this era^8^. However, GVHD after LT is relatively rare; thus, the risk factors, and treatments have not yet been well studied. Some studies have shown a relationship between HLA compatibility and GVHD in LDLT. A study by Chan et al. evaluated several risk factors including HLA mismatches, and found no significant influence on GVHD^3^. However, this study did not evaluate HLA mismatch types, such as the D → R one-way mismatch. Other studies have focused on one-way HLA mismatches in LDLT. Kiuchi et al*.* reported four patients with GVHD who all had complete HLA mismatch^9^. Kamei. et al*.* showed four GVHD patients (44%) among nine cases of D → R one-way MM3^7^. This study also showed eight reported GVHD cases, of which seven cases had D → R one-way MM3, while one case had D → R one-way mismatch in HLA-A and–DR loci with identical HLA-B. Another study, in Japan, evaluated 346 pairs of LDLT and revealed three cases (60%) of GVHD among five D → R one-way MM3^6^. These reports support our result that D → R one-way MM3 is a risk factor for GVHD. D → R one-way MM3 should be carefully checked from the LT preparation process. It is especially important when donor's HLA loci are homozygous because blood-related recipients shared one-haploid identical HLA with donors. Although pre-operative evaluation of HLA mismatch and forbidding D → R one-way MM3 is not generally established in guidelines and transplantation centers because of the cost-effectiveness and rarity of GVHD, it is time to consider detailed evaluation to avoid the possibility of fatal GVHD^10^. The reason why two cases (except one who received early re-LT) with D → R one-way mismatch at HLA-A, B and DR did not developed GVHD is unknown. One recent study hypothesized the possibility of non-one-way mismatch in HLA-C, DQ or DP may influenced the consequences^11^. This study also showed three cases who did not developed GVHD among six cases with D → R one-way mismatch at HLA-A, B and DR (50%). However, two cases of this study died early after LT (at 35 and 54 days) due to other causes, and HLA-C, DQ and DP of one survived case was not evaluated. Our study also did not evaluate the HLA-C, DQ and DP because this diagnostic tool was applied in 2022 in our center. The reason why some of patients with D → R one-way mismatch at HLA-A, B and DR did not suffer GVHD is not well-known and maybe influenced by the following risk factors of next paragraph. The previous studies about D → R one-way MM in LDLT are summarized in Table 7. Majority of GVHD cases had D → R one-way MM3 (HLA-A, B and DR) with homozygous donors. However, there are some reported GVHD cases without D → R one-way MM at 3 loci (HLA-A, B and DR). Shimizu, et al. reported a GVHD case with D → R one-way MM HLA-B, DR and identical HLA-A^12^. Yu, et al. also reported a GVHD case with D → R one-way MM at HLA-A, B and mismatch at one allele of HLA-DR, whose donor was not homozygous at HLA-DR^13^. Shimata, et al. reported a GVHD case with D → R one-way MM at HLA-A, B and C but not -DR^14^.

**Table 7:** Previous studies about HLA one-way mismatches and GVHD after liver transplantation.

References	Country	Type of LT	GVHD No./Total No	Donor against recipient one-way MM of HLA		Mortality (%)
				in GVHD patients/Donor homozygosity	in total patients	
Kiuchi^9^	Japan	LDLT	1/280	1 case: one-way MM at 6 loci (A, B, C, DR and DQ)/ Donor: homozygous at A, B, C, DR and DQ	MM at 3 loci (A, B and DR): 4 cases (1 GVHD case with MM at A, B, C, DR and DQ)	1 (100%)
Nemoto^24^	Japan	LDLT	1 (Case report)	1 case: one-way MM at 6 loci (A, B, C, DR and DQ)/Donor: homozygous at A, B, C, DR and DQ	-	0
Seojima^25^	Japan	LDLT	1 (Case report)	1 case: one-way MM at 3 loci (A, B and DR)/Donor: homozygous at A, B and DR	-	1 (100%)
Kamei^7^	Japan	LDLT	8/906	8 cases: one-way MM at 3 loci (A, B and DR) (1 case had minor heterogeneity at 2nd field allele of B)/All donors: homozygous at A, B and DR	MM at 3 loci (A, B and DR): 9 cases (4 GVHD cases) MM at 2 loci: 26 cases (no GVHD case) MM at 2 loci: 171 cases (no GVHD case)	8 (100%)
Shimizu^12^	Japan	LDLT	1 (Case report)	1 case: one-way MM at 2 loci (B, DR) and identical at A/ Donor: homozygous at A, B and DR	-	1 (100%)
Uchiyama^6^	Japan	LDLT	3/390	3 cases: one-way MM at 3 loci (A, B and DR)/All donors: homozygous at A, B and DR	MM at 3 loci (A, B and DR): 5 cases (3 GVHD cases) MM at 2 loci: 1 case (no GVHD case) MM at 1 locus: 2 cases (no GVHD case)	3 (100%)
Yu^13^	South Korea	LDLT	1 (Case report)	1 case: one-way MM at 2 loci (A, B) and MM at 1 allele of HLA-DR/ Donor: homozygous at A, B but not at DR	-	1 (100%)
Shimata^14^	Japan	LDLT	1 (Case report)	1 case: one-way MM at 3 loci (A, B, C) but not at DR/ Donor: homozygous at A, B, C but not at DR	-	1 (100%)
Ghandora^10^	Saudi Arabia	LDLT	1 (Case report)	1 case: one-way MM at 6 loci (A, B, DR, DQ and DP)/Donor: homozygous at A, B, DR, DQ and DP	-	0 (100%)
Hirata^11^	Japan	LDLT	4/1759	1 case: one-way MM at 3 loci (A, B and DR) 2 cases: one-way MM at 6 loci (A, B, C, DR, DQ and DP)/3 donors: homozygous at 3 loci (2 cases at 6 loci) 1 case: no one-way MM at all 6 loci/Donor: no heterozygous alleles	MM at 3 loci (A, B and DR): 6 cases (3 GVHD cases) MM at 2 loci: 41 cases (no GVHD case) MM at 1 locus: 9 cases (no GVHD case)	4 (100%)

GVHD, graft versus host disease; HLA, human leukocyte antigen; LDLT, living donor liver transplantation; MM, mismatches.

There are also other risk factors for GVHD in LT. The reported risk factors of GVHD other than HLA one-way mismatch are usually based on DDLT era. Our study showed four GVHD cases who received liver from deceased donor without D → R one-way MM3. Other reports also shows similar GVHD cases in DDLT era which means there are influence other than HLA one-way mismatch^15^. One study reported that glucose intolerance, either type I DM or type II DM, is a risk factor for GVHD^3^. This study also introduced autoimmune hepatitis or HCC recipients receiving a steatotic donor liver, showing a risk of GVHD development. HCC is known to cause immune system dysfunction and may increase the risk of GVHD^2^. In contrast, GVHD may occur less frequently in recipients with HCV, which is known to inhibit T-cell mediated signaling pathways. However, there was no significant relationship between DM, HCC, or HCV infection and GVHD development in either LDLT or DDLT of our study population.

Some studies have shown age difference as a risk factor for GVHD after DDLT. The greater the age difference between the recipient and donor, the higher the risk of GVHD, especially when the difference is > 20 years^4,5^. In our study, age difference more than 20 years showed HR of 1.79 for GVHD incidence without statistical significance in univariate analysis (*p* = 0.409) in whole patients. Neither analysis of age difference on DDLT patient did not show statistical difference (*p* = 0.475).

Re-transplantation showed a significantly higher risk of GVHD in univariate analysis (HR 5.85, *p* = 0.028) in our study. Among 74 patients who received re-LT, two patients (2.7%) developed GVHD, while seven patients (0.5%) developed GVHD among 1,313 recipients who received first LT. However, this re-LT showed only a trend of GVHD risk in multivariate analysis (HR 5.21, *p* = 0.094), possibly due to the small number of GVHD cases. When applied only to DDLT patient, re-LT showed a small tendency of GVHD risk in univariate analysis (HR 5.26, *p* = 0.97). Another non-human study which analyzed HLA one-way mismatched rat, suggest the amount of transfused donor lymphocyte may influence the GVHD development^16^. These risk factors of GVHD needs more future research.

The relationship between HLA mismatch and graft survival in LDLT is still controversial, while HLA compatibility has a significant impact on graft survival in kidney or heart transplants^17^. Victor et al*.* showed that recipients with no HLA mismatch had higher 5-year graft failure (19.3%) in much a larger group of 29,675 LT cases, including living donor and partial organ transplants in the Organ Procurement and Transplant Network (OPTN) database of the USA^18^. This study showed no statistical relationship between HLA zero-mismatch and graft failure when living donors and partial organ transplants were excluded. However, a follow-up study of this group using 631 LDLT cases from the OPTN database showed no relationship between HLA matching and graft survival. However, these two studies did not analyze the type of HLA mismatch and did not focus on one-way mismatches. The reason why the D → R one-way MM3 increased the risk of graft failure in our study is not known. Previous studies on D → R one-way MM3 have focused only on the relationship with GVHD. However, our study included only two patients with graft failure among patients with D → R one-way MM3, which may limit the power of the statistical analysis.

The usual clinical manifestations of GVHD after LT are rash, GI symptoms, and cytopenia. Suspicion of and diagnostic procedures for GVHD were often delayed because many GVHD patients were already taking multiple drugs, such as antibiotics and ganciclovir, which may induce cytopenia or rash as side effects. Diarrhea is also not a rare symptom in LT patients during the first 2 months after LT^19^. In our report, the median gap between the first onset and diagnosis was 4.5 days with two patients showing a longer gap of more than 10 days. This might have resulted in late initiation of treatment. The three major signs of GVHD, rash, cytopenia and GI symptoms, should be observed closely, especially when patients show one of the symptoms around 1 months after LT. Immediate diagnostic procedures such as skin biopsy, colonoscopic biopsy, or qPCR analysis, should be performed when GVHD is suspected. Because cytopenia is caused by many drugs, bone marrow biopsy should be done with caution in patients with cytopenia. However, the first onset of GVHD may appear far more than 1 month after LT, such as in case #2 and #8 (postoperative day 60 and 80, respectively). Some articles have reported that acute GVHD may appear after approximately 90 days after LT^2,15^.

While histologic confirmation may add accuracy for diagnosing GVHD, it is often misdiagnosed and weakly correlated to clinical course. The pathologic diagnostic criteria are still under discussion^20^. The traditional definitive method for confirming GVHD, using deoxyribonucleic acid (DNA) is a PCR targeting short tandem repeats (STRs). Three patients (Case #6–8) in our study were assessed for GVHD using a qPCR method targeting INDEL, which was recently introduced. The donor DNA fractions of the recipient peripheral blood were 39.7%, 78.4%, and 4.8% in cases #6, #7, and #8, respectively. Two patients with a high donor DNA fraction died due to aggravation of GVHD, while case #8, with a relatively low donor DNA fraction, showed mild symptoms without rash and was still alive for 370 days. Also, case #8 was the only GVHD patient among LDLT patients whose HLA typing was not a D → R one-way MM3. It may be assumed that a relatively low degree of macrochimerism leads to a better prognosis. However, only a few articles have shown the relationship between the degree of macrochimerism and patient survival. One study reported that among seven LT patients with GVHD, five patients who had more than 10% macro-chimerism died, while half of the patients with less than 10% macrochimerism survived (one patient with 8% macrochimerism survived and one patient with 4% died)^21^. DNA was detected by qPCR and flow cytometry using sequence-specific primers. Further research is needed to evaluate the relationship between the degree of chimerism and prognosis.

The treatment of GVHD after LT has not yet been established. High-dose steroids, which show favorable outcomes for GVHD after hematopoietic stem cell transplantation, are frequently used in LT, showing only disappointing result^2^. ATG, an interleukin-2 antagonist regimen, was also attempted but resulted in a mortality rate of more than 70%. Although TNF-α inhibitors showed survival gain in a previous report, they did not show any benefit in our study (cases #6 and #7)^22,23^. However, these agents were administered after the patients already developed septic shock and may not have had sufficient time to efficiently treat GVHD. The treatment of GVHD in LT requires further investigation.

Our study was limited by the small number of GVHD cases, thus the validity of the statistical conclusion was relatively low. This small number was due to the characteristic rarity of GVHD. Our study could not analyze the type of HLA-C, DQ and DP. Diagnosis of GVHD mainly depends on pathological diagnosis while only three patients were diagnosed by qPCR method. However, this study has many data of HLA relationships between donors and recipients, including 994 LDLT and 393 DDLT patients, which in turn showed the possible effect of D → R one-way MM3 on GVHD.

In conclusion, the D → R one-way mismatch at three loci greatly increased the incidence of GVHD after LT, leading to patient death. The mortality rate of GVHD is very high, and treatment is still not well established. Preoperative HLA evaluation and avoidance of GVHD are recommended.

## Supplementary Information

Supplementary Information.

## Data Availability

The datasets used and/or analysed during the current study are available from the corresponding author on reasonable request.

## Abbreviations

ABO-I: ABO-incompatible
ATG: Anti-thymocyte globulin
BMI: Body mass index
CMV: Cytomegalo virus
DDLT: Deceased donor liver transplantation
DM: Diabetes mellitus
DNA: Deoxyribonucleic acid
D → R one-way MM3: Donor against recipient one-way mismatch at three loci
GI: Gastrointestinal
GVHD: Graft versus host disease
HBV: Hepatitis B virus
HCV: Hepatitis C virus
HCC: Hepatocellular carcinoma
HLA: Human leukocyte antigen
HR: Hazard ratio
HTN: Hypertension
LDLT: Living donor liver transplantation
LT: Liver transplantation.
MELD: Model for end-stage liver disease
PCR: Polymerase chain reaction
OPTN: Organ procurement: and transplant network
qPCR: Quantitative polymerase chain reaction
STRs: Short tandem repeats
