# Author Correction: The clinical impact of donor against recipient HLA one way mismatch on the occurrence of graft versus host disease in liver transplantation

**DOI:** 10.1038/s41598-022-27044-7

**Published:** 2022-12-30

**Authors:** Sang Jin Kim, Sunghae Park, Jinsoo Rhu, Jong Man Kim, Gyu-Seong Choi, Jae-Won Joh

**Affiliations:** 1 Department of Surgery, Korea University College of Medicine, Seoul, Republic of Korea; 2 Division of Hepatobiliopancreas and Transplant Surgery, Korea University Ansan Hospital, Ansan, Republic of Korea; 3 Department of Surgery, Samsung Medical Center, Sungkyunkwan University School of Medicine, 81 Irwon-Ro, Gangnam-Gu, Seoul, 06351 Republic of Korea

Correction to: *Scientific Reports* 10.1038/s41598-022-24778-2, published online 25 November 2022

The original version of this article contained an error in the spelling of the author Gyu-Seong Choi which was incorrectly given as Gyu Seung Choi.

The original article has been corrected.

