# Supplementary material for: The clinical impact of donor against recipient HLA one way mismatch on the occurrence of graft versus host disease in liver transplantation
Source: Sci Rep. 2022 Nov 25;12:20337. doi: 10.1038/s41598-022-24778-2 (PMC9700759; doi:10.1038/s41598-022-24778-2)
Supplement: Supplementary file 1 — Supplementary Information. [file 41598_2022_24778_MOESM1_ESM.docx]

**Supplement 1**. HLA mismatch types

| Mismatch relationship | | All patient (n=1387) | LDLT (n=994) | DDLT (n=393) |
| --- | --- | --- | --- | --- |
| Donor against recipient  total mismatch numbers | 0 | 48 (3.5%) | 48 (4.8%) | 0 |
|  | 1 | 91 (6.6%) | 90 (9.1%) | 1 (0.3%) |
|  | 2 | 257 (18.5%) | 243 (24.4%) | 14 (3.6%) |
|  | 3 | 426 (30.7%) | 387 (38.9%) | 39 (9.9%) |
|  | 4 | 160 (11.5%) | 71 (7.1%) | 89 (22.6%) |
|  | 5 | 222 (16.0%) | 85 (8.6%) | 137 (34.9%) |
|  | 6 | 183 (13.2%) | 70 (7.0%) | 113 (28.8%) |
| Donor against recipient  total mismatch category | 0-1 | 139 (10.0%) | 138 (13.9%) | 1 (0.3%) |
|  | 2-3 | 683 (49.2%) | 630 (63.4%) | 53 (13.5%) |
|  | ≥ 4 | 565 (40.7%) | 226 (22.7%) | 339 (86.3%) |
| Recipient against donor  total mismatch numbers | 0 | 44 (3.2%) | 44 (4.4%) | 0 |
|  | 1 | 98 (7.1%) | 95 (9.6%) | 3 (0.8%) |
|  | 2 | 251 (18.1%) | 240 (24.1%) | 11 (2.8%) |
|  | 3 | 420 (30.3%) | 386 (38.8%) | 34 (8.7%) |
|  | 4 | 168 (12.1%) | 74 (7.4%) | 94 (23.9%) |
|  | 5 | 223 (16.1%) | 85 (8.6%) | 138 (35.1%) |
|  | 6 | 183 (13.2%) | 70 (7.0%) | 113 (28.8%) |
| Recipient against donor  total mismatch category | 0-1 | 142 (10.2%) | 139 (14.0%) | 3 (0.8%) |
|  | 2-3 | 671 (48.4%) | 626 (63.0%) | 45 (11.5%) |
|  | ≥ 4 | 574 (41.4%) | 229 (23.0%) | 345 (87.8%) |

Abbreviations: LDLT, living donor liver transplantation; DDLT, deceased donor liver transplantation.

**Supplement 2.** HLA types of donor against recipient one-way mismatch at three loci cases.

|  | Recipient HLA | | | | | | Donor HLA | | | | | | Occurrence of GVHD | Relationship  (Donor) |
| --- | --- | --- | --- | --- | --- | --- | --- | --- | --- | --- | --- | --- | --- | --- |
| Case no. | A |  | B |  | DR |  | A |  | B |  | DR |  |  |  |
| #1 | 02 | 33 | 44 | 54 | 13 | 15 | 33 | - | 44 | - | 13 | - | Yes | Mother |
| #2 | 02 | 33 | 35 | 44 | 4 | 7 | 33 | - | 44 | - | 07 | - |  | Son |
| #3 | 24:02 | 33:03 | 15:07 | 44:03 | 07:01 | 12:02 | 33:03 | - | 44:03 | - | 07:01 | - |  | Daughter |
| #4 | 24:02 | 33:03 | 44:03 | 46:01 | 07:01 | 08:03 | 33:03 | - | 44:03 | - | 07:01 | - | Yes | Daughter |
| #5 | 31:01 | 33:03 | 44:03 | 46:01 | 08:03 | 13:02 | 33:03 | - | 44:03 | - | 13:02 | - | Yes | Daughter |
| #6 | 24:02 | 33:03 | 44:03 | 59:01 | 04:05 | 13:02 | 33:03 | - | 44:03 | - | 13:02 | - |  | Sister |
| #7 | 11:01 | 33:03 | 54:01 | 58:01 | 04:05 | 13:02 | 33:03 | - | 58:01 | - | 13:02 | - | Yes | Daughter |

Abbreviations: HLA, human leukocyte antigen; GVHD, graft versus host disease.

* Case#2 developed primary non-function of the graft and received re-LT on postoperative day 4, after which the HLA relationship changed.

* HLA types of Case#1 and 2 were not evaluated to the four digits allele level.

**Supplement 3.** Risk factors of GVHD according to LDLT and DDLT.

|  | LDLT | | DDLT | |
| --- | --- | --- | --- | --- |
| Risk factors | Univariate analysis | | Univariate analysis | |
|  | HR (95% CI) | P-value | HR (95% CI) | P-value |
| D→R one-way MM3^*^ | - | - | No case | - |
| Donor age | 1.02 (0.96-1.08) | 0.619 | 0.98 (0.93-1.04) | 0.473 |
| Age difference  (Recipient - donor age) | 0.99 (0.94-1.04) | 0.606 | 1.04 (0.98-1.09) | 0.206 |
| Age difference ≥ 20 year | 36.2 (0.01-1.0E+4) | 0.377 | 0.49 (0.07-3.48) | 0.475 |
| Age | 1.01 (0.94-10.7) | 0.883 | 1.04 (0.96-1.13) | 0.322 |
| Male | 0.23 (0.04-1.38) | 0.108 | 45.9 (0.01-1.8E+5) | 0.366 |
| BMI | 0.96 (0.76-1.20) | 0.691 | 0.93 (0.77-1.12) | 0.428 |
| CTP score | 1.12 (0.80-1.57) | 0.505 | 0.94 (0.59-1.50) | 0.808 |
| MELD score | 1.02 (0.94-1.10) | 0.707 | 1.04 (0.94-1.16) | 0.405 |
| HTN | 4.22 (0.70-25.2) | 0.115 | 0.04 (0.00-2.1E+4) | 0.634 |
| DM | 1.03 (0.12-9.25) | 0.977 | 3.81 (0.54-27.1) | 0.181 |
| Re-LT | 0.05 (0.00-1.56E+20) | 0.905 | 5.26 (0.74-37.1) | 0.097 |
| ABO incompatible | 0.04 (0.00-584) | 0.502 | No case | - |
| HBV | 0.39 (0.06-2.33) | 0.302 | 0.50 (0.08-2.97) | 0.442 |
| HCV | 0.05 (0.00-6.3E+5) | 0.713 | 0.05 (0.00-2.5E+6) | 0.734 |
| Alcoholic | 1.51 (0.17-13.5) | 0.714 | 3.01 (0.42-21.4) | 0.270 |
| HCC | 0.98 (0.16-5.86) | 0.981 | 2.36 (0.33-16.8) | 0.390 |

*: D→R one-way MM3 in LDLT was unable to analyze in cox regression due to small number of cases (D→R one-way MM3: 7 cases, GVHD: 5 cases).

**Supplement 4**. Clinical courses of each GVHD patients.

Case #1. Patient with periportal biloma who experienced rapid progression of GVHD.

A 49-year-old male received DDLT for HBV-related liver cirrhosis and HCC. The patient received only steroid pulses without basiliximab as an induction immunotherapy. The patient was discharged from the hospital on post-operative day (POD) 17 without any specific events. However, he was re-admitted due to fever, and periportal biloma was found on CT and centrilobular necrosis on pathologic examination. He received antibiotics and percutaneous catheter drainage insertion on the biloma on POD 20; however, a rash appeared on his flank on POD 24. On POD 6, the patient developed a fever and pancytopenia. Skin biopsy revealed GVHD. On the same day, cyclosporine was changed to tacrolimus, and prednisolone 20 mg was administered twice daily. Anti-thymocyte globulin (ATG) was also added for 3 days, and plasmapheresis was initiated. However, he died of septic shock on POD 31.

Case #2. GVHD extended from the colon and skin to esophagus after 40 days.

A 20-year-old female received LDLT for drug-induced acute hepatic failure. The donor was her mother, and a right liver graft was donated. Ganciclovir was started on POD 28 because of CMV antigenemia. Subhepatic fluid collection was observed on POD 34, which was followed by percutaneous catheter drainage (PCD) insertion. Rash developed on the extremities and face on POD 60 and fever and diarrhea on POD 61. Skin and colonoscopic biopsies were performed on POD 63, revealing GVHD on both sides. MMF was administered and methylprednisolone was tapered from 8 mg to 4 mg twice daily. Cyclosporine was maintained with supportive therapy for diarrhea and nutrition. Diarrhea improved slowly over one month, and she received an endoscopic retrograde biliary drainage (ERBD) stent due to possible biliary obstruction on POD 84. With a slight improvement in gastrointestinal symptoms, MMF was restarted on POD 92. However, leukopenia and vomiting developed on POD 100, followed by a pathological diagnosis of GVHD on the esophagus on POD 104. Although cyclosporine and MMF were stopped, and hydrocortisone 200 mg daily was injected for septic shock, her condition worsened rapidly, and she died on POD 110.

Case #3. Delayed detection of GVHD due to CMV pneumonia treatment

A 53-year-old male received DDLT for alcoholic LC. After discharge on POD 23 without a specific event, he was re-admitted due to pancytopenia and elevated C-reactive protein (CRP) levels on POD 38. Ganciclovir was administered on POD 41 because of CMV antigenemia and pulmonary infiltration. Mild diarrhea developed on POD 43, and CMV pneumonia was confirmed by inclusion bodies from transbronchial lung biopsy at POD 45. His condition worsened with a high fever (POD 47), whole-body rash (POD 48), and massive diarrhea (POD 49). Although tacrolimus was discontinued, followed by hydrocortisone 300 mg daily with broad-spectrum antibiotics, the patient died on POD 52. Skin biopsy showed GVHD and focal endothelial CMV infection after death.

Case #4. Second episode of GVHD involving whole gastrointestinal organs.

A 64-year-old male received re-DDLT because of failure of a previously received liver graft due to chronic rejection. The first LT was LDLT performed 2 years ago because of HCV, LC, and HCC. A skin rash and intermittent fever developed on POD 23. Tacrolimus was stopped owing to fever. Owing to sustained fever, skin rash, and hypotension, a biopsy was performed on POD 27. Daily 25 g of intravenous immunoglobulin G (IVIG) was administered daily for 2 days and methylprednisolone (MPD) 60 mg daily for 7 days. This was followed by the restart of tacrolimus and 4 mg MPD twice a day from POD 34. After a short recovery from symptoms, pancytopenia developed 20 days after the first onset (POD 43), followed by diarrhea (POD 51). Colonoscopic examination revealed no specific abnormality in the sigmoid colon; however, random biopsy revealed GVHD. During conservative treatment, the patient experienced abdominal pain, vomiting, fever, and leukopenia on POD 63. An additional 25 mg of IVIG (for 3 days) and 60 mg MPD (continuous) were administered when the patient developed septic shock on POD 65. However, the patient’s condition worsened, and he died on POD 74. The CT image on POD 71 showed diffuse layered wall thickening from the stomach to the sigmoid colon, compatible with GVHD.

Case #5. Early-onset GVHD and rapid aggravation

A 61-year-old male received re-DDLT for graft failure due to biliary complications. The first LT was ABO-incompatible LDLT performed 1 year ago because of HBV and HCC. Due to cholangiohepatitis and candidemia, broad-spectrum antibiotics and antifungal agents were administered before LT. WBC count decreased to within the normal range on POD 17, followed by pancytopenia on POD 24 with diarrhea, which was misunderstood as a side effect of drug prescription. However, a rash developed on both buttocks on POD 25, and a skin biopsy was performed on POD 30, revealing GVHD. Although hydrocortisone 200 mg was started daily from POD28 for 8 days, he died on POD 42.

Case #6. Bile leakage and IV ganciclovir disrupted GVHD diagnosis

A 66-year-old male received LDLT for HBV-related LC and HCC. Donor was her daughter. Due to variations in the bile duct (two right anterior hepatic ducts and one right posterior hepatic duct), bile leaked on POD 2 with well-functioning Jackson-Pratt drainage. IV ganciclovir was started because of CMV infection on POD 21. Temporary diarrhea (POD 23), a mild rash (POD 24), and decreasing WBC count (POD 25) developed serially. While the daily bile leak amount was 600–800 ml, a temporary ERBD stent was inserted through endoscopic retrograde cholangiopancreatography (ERCP) on POD 28. However, leukopenia was aggravated, with a WBC count of 670 count/μL on the same day, and tacrolimus and MMF were stopped. A skin biopsy on POD 29 revealed GVHD. A qPCR chimerism assay was performed, resulting in a donor DNA fraction of 39.68%. Although steroid pulse therapy (500 mg one day and scheduled tapering) with 25 mg etanercept was initiated, the TNF-α inhibitor was started on POD 30 and the patient died the next day.

Case #7. High donor DNA fraction with macro-chimerism

A 49-year-old male received DDLT for alcoholic LC. He was discharged on POD 22 without any specific abnormality. The patient developed fever on POD27 and pancytopenia on POD 29. He was re-admitted to the hospital on POD 31 and received oseltamivir for a positive influenza B virus test. Diarrhea developed on POD 33 and a whole-body rash on POD34. Tacrolimus was stopped on POD 35, and skin biopsy was performed on POD 37. Upon suspicion of GVHD, hydrocortisone 200 mg bolus injection was followed by 32 mg twice a day. Although additional etanercept 25 mg was injected on POD 38, the patient died the next day. Pathologic reports showed GVHD, and qPCR showed a donor DNA fraction of 78.38%.

Case #8. Relatively low donor DNA fraction with late onset and mild symptom

A 69-year-old female received LDLT for HBV-related HCC. The donor was her son, and HLA type did not show D→R one-way mismatches. After discharge on POD 23, she was re-admitted for the treatment of CMV gastritis from POD 33 to 56. She stayed at home taking valganciclovir until she felt a heart-burning sensation and vomited on POD 80, showing thrombocytopenia at the emergency department. Pancytopenia developed on POD 81, and esophagoduodenoscopy (EGD) showed healing of CMV gastritis. Therefore, the antiviral agent was discontinued. Owing to hematochezia and diarrhea on POD 84, sigmoidoscopy was performed, showing nonspecific colitis, which was later diagnosed as GVHD in the pathologic report. MPD (60 mg of MPD injected twice daily for 1 week from POD 90. Tacrolimus and everolimus were stopped for 3 days and tacrolimus was restarted at the lowest dose (0.25 mg daily). qPCR showed macro-chimerism; however, the proportion of donor DNA was relatively low (4.76%). Cytopenia, diarrhea, and nausea improved after steroid treatment, whereas platelets improved very slowly after POD 101 and reached a normal range approximately 10 months postoperatively. The patient continued to live for 370 days and visited on the scheduled outpatient department appointments.

Case #9. Aggravation of GVHD with combined liver abscess

A 41-year-old male received LDLT for alcoholic LC. The patient’s daughter was a donor. Total bilirubin level increased on POD 11 with possible biliary anastomosis site stricture on CT scan, which was treated with ERBD stent insertion on POD 16. However, due to intrahepatic abscess at liver segments 5/8 and intrahepatic duct dilatation, PCD and percutaneous transhepatic bile drainage were performed on POD 34. Tacrolimus was stopped owing to bacteremia. Rash developed on POD 36, and 100 mg hydrocortisone was started twice a day since POD 38 because of the septic state. A skin biopsy was performed on POD 41, which revealed GVHD followed by pancytopenia on POD 49. The patient’s condition worsened, and he died on POD 56.

Case #10. Missing of GVHD detection due to severe CMV proctitis.

A 66-year-old female received LDLT for HBV-related HCC. The patient’s daughter was the donor. The patient developed diarrhea on POD 27 and leukopenia on POD28. Clostridium difficile was detected in the stool, followed by vancomycin and metronidazole treatment. Due to hematochezia at POD#33, MPD 60mg was injected daily upon suspicion of GVHD. Tacrolimus and MMF was also stopped. On sigmoidoscopy at the next day (POD 34), severe inflammation of the mucosa, blood, and dirty exudate were found in the rectum and sigmoid colon, with probable multiple wall defects in the sigmoid colon. The pathological report of the rectum showed highly suspicious CMV proctitis, while CMV antigen was not detected in the serum. After excluding the possibility of GVHD, the dose of MPD was reduced to 16 mg twice daily and ganciclovir was administered. However, the patient’s condition worsened, with sepsis and skin rash developing on POD42. The patient died on POD 45, and the pathologic result of skin reported GVHD.
